# Assessment of Presence and Metastatic Involvement of Lymph Nodes in Anterior Periprostatic Fat (APPF) in Prostate Cancer Patients Treated with Robotic and Laparoscopic Radical Prostatectomy

**DOI:** 10.3390/jcm15124614

**Published:** 2026-06-14

**Authors:** Mudassir Wani, Jayasimha Abbaraju, Bikram Bhattacharjee, Abdousamad Said Omar, Hasan Al-Chalabi, Sanjeev Madaan

**Affiliations:** 1 Department of Urology, Cardiff and Vale University Health Board, Cardiff CF14 4XW, UK; mudassir.wani@nhs.net; 2 Faculty of Social and Applied Sciences, Canterbury Christ Church University, Chatham ME4 4UF, UK; 3 Department of Urology & Nephrology, Dartford and Gravesham NHS Trust, Dartford DA2 8DA, UK; jayasimha.abbaraju@nhs.net; 4 Department of Radiology, Dartford and Gravesham NHS Trust, Dartford DA2 8DA, UK; bikram.bhattacharjee@nhs.net; 5 Department of Surgery, Aneurin Bevan University Health Board, Newport NP18 3XQ, UK; abdousamad.omar@wales.nhs.uk; 6 Department of Urology, Aneurin Bevan University Health Board, Newport NP18 3XQ, UK; hasan.al-chalabi@wales.nhs.uk

**Keywords:** anterior periprostatic fat, lymph nodes, radical prostatectomy

## Abstract

**Introduction**: Lymph nodes (LN) in the anterior periprostatic fat (APPF) may harbour metastatic disease in patients with Prostate Cancer (PCa). We investigated the incidence and significance of LN in APPF tissue removed during robotic and laparoscopic radical prostatectomy (RP). **Patients and Methods**: We retrospectively analysed RP performed by a single surgeon from 2013 to 2023. A total of 670 patients underwent RP, with 407 procedures conducted laparoscopically and 263 robotically. Histological results were available for 509 patients, who were examined for the presence of LN and any evidence of metastatic involvement. **Results**: LN were detected in the periprostatic fat of eighty patients; however, only twelve had lymph node metastasis. Seven of the twelve patients presented with prostate-specific antigen (PSA) levels greater than 10 ng/mL. All LN-positive patients had a Gleason score of seven or higher. On MRI, all patients had a PIRADS score of four or higher, and eleven were staged at T3 or higher. Additionally, all twelve patients had a Briganti score exceeding twenty. **Conclusions**: Our series indicates that the APPF contains LN that can harbour metastatic disease. Patients can have LN involved in APPF without the involvement of pelvic LN. Therefore, our study suggests that routine excision of APPF should be considered for appropriate LN staging and to avoid missing any metastasis, and that scoring systems like Briganti should be used to help identify this high-risk group.

## 1. Introduction

Prostate cancer (PCa) remains the second most common malignancy and the fifth leading cause of cancer-related mortality in men [1]. It is estimated that around 80% of men diagnosed with PCa have either localised or locally advanced disease. Management options for these patients include active surveillance, surgery, radiotherapy alone, or radiotherapy combined with androgen deprivation therapy, with satisfactory outcomes [2]. One of the most significant poor prognostic factors in PCa is lymph node metastasis (LNM) [3]. The presence of LNM (pathological N1 disease) indicates a worse prognosis, being associated with higher risks of recurrence and reduced cancer-specific survival. Identifying nodal involvement influences postoperative management, as this group of patients often receives adjuvant therapies (such as hormonal therapy or radiation) that can improve treatment outcomes [4]. As a result, lymph node staging is crucial for treatment planning and follow-up management.

Historically, APPF has been discarded during RP. However, three recent studies have shown that lymph nodes are identified in 9–17% of APPF specimens, with a metastatic rate of up to 15% [4,5,6]. Evidence indicates that APPF LN can be positive for metastasis, even when pelvic lymph nodes in the same patient are negative [6]. These findings collectively suggest that although most patients do not have lymphatic tissue in the APPF, a non-negligible minority do, and a very small fraction may have cancer spread limited to this area. Despite this, APPF evaluation remains inconsistently adopted, and its clinical significance in robotic and laparoscopic RP is poorly defined. By integrating APPF evaluation into standard surgical practice, clinicians can achieve more accurate staging, tailor postoperative management, and ultimately improve patient outcomes.

The primary aim of the study was to quantify the incidence of APPF LN and its metastatic potential in a large cohort of RP patients, and the secondary aim was to correlate APPF metastasis with high-risk clinicopathological features.

## 2. Material and Methods

This retrospective study analysed radical prostatectomies (RP) performed by a single surgeon (SM) from 2013 to 2023 to review APPF histology.

The first stage of data collection was to retrieve data on all patients who underwent RP, either laparoscopic or robotic, between 2013 and 2023 under the named consultant. The theatre help desk provided the data after completing the necessary documentation and obtaining the required permissions. After reviewing the patient details, the electronic health records were retrieved for all patients. We included all patients with complete clinical information, including PSA, pelvic and prostate MRI, prostate biopsy results, and post-procedure histopathology results. The exclusion criteria included any missing clinical information or investigation results, as well as patients who had received neo-adjuvant therapy or prior radiation.

We reviewed the surgical technique and found no difference in the approach to APPF removal between laparoscopic and robotic prostatectomy. The key steps included direct visualisation after placement of the camera, trocar, and port; release of bowel adhesions if required; and division of the medial and median umbilical ligaments to access the space of Retzius. The prostate was identified, and periprostatic fat was removed. Electrocautery was used to control bleeding. The specimen was retrieved and sent separately for histopathology.

Patients with lymph nodes in APPF were assessed for Prostate-Specific Antigen (PSA) levels, Gleason scores, MRI staging, PIRAD, and Briganti scores.

## 3. Results

**Figure 1:**
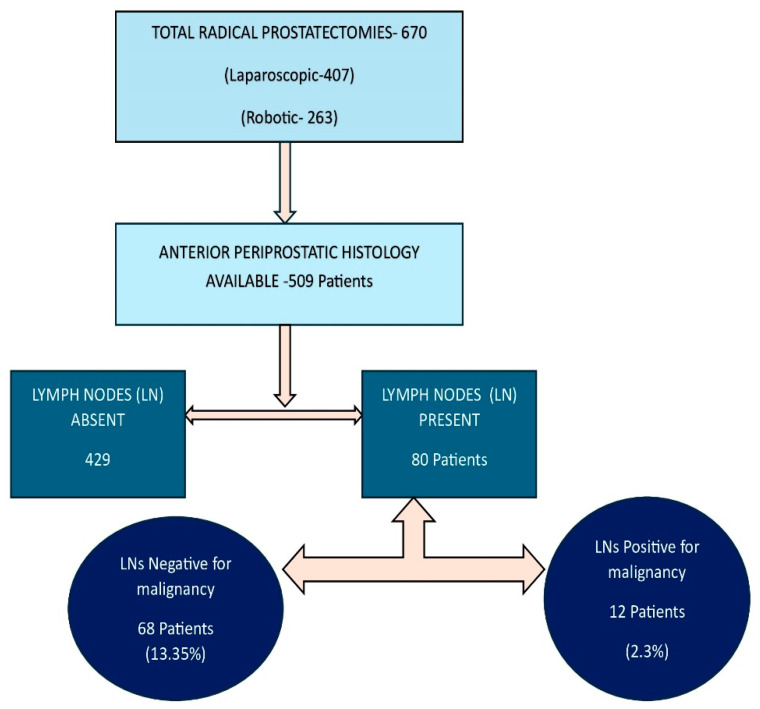
Patient flow chart.

A total of 670 patients underwent radical prostatectomy (RP), of whom 407 (61%) underwent a laparoscopic procedure and 263 (39%) a robotic approach. Anterior periprostatic fat (APPF) was histologically evaluated in 509 (76%) patients, while pelvic lymph node dissection (PLND) was performed in 226 (34%). In the 509 patients with APPF evaluation, 80 (16%) had lymph nodes in the excised fat, of whom 12 (2.3%) were malignant (Figure 1: Patient Flow Chart).

Full details on PSA distribution, MRI findings, Gleason, tumour stage, and Briganti score [7] in APPF-positive vs. APPF-negative groups are provided in Table 1.

Results revealed that APPF LN positivity was commonly associated with higher PSA categories, advanced stage (T3a or T3b), higher Gleason (≥8), and elevated Briganti scores (>20).

The results of APPF LN positive twelve patients are as follows:

1. PSA: 7 patients (58%) had PSA > 10 ng/mL.
2. Gleason Score: All patients had a Gleason score of 7 or more; 5 patients had a Gleason Score of 8 or higher.
3. PIRAD score: All patients had a PIRAD score of 4 or more.
4. MRI staging: revealed T3a or T3b on MRI contributed the highest fraction of APPF nodal metastases, 11 (92%). Conversely, T2a or T2b tumours rarely showed APPF involvement.
5. Briganti scoring revealed that all patients with positive APPF lymph nodes had a score of more than 20. Scores exceeding 50 were especially notable, as 7 (58%) patients in this metastatic subset fell into that range.

Regarding the nodal burden in APPF, most patients with node-positive APPF harboured only one lymph node.

## 4. Discussion

The prostate is surrounded by a fat depot, often referred to as periprostatic adipose tissue (PPAT). PPAT, due to its paracrine action, is considered to cause PCa progression [8]. Anterior periprostatic fat (APPF), which represents PPAT in direct contact with the prostate, has also been found to contain lymph nodes that can harbour metastatic deposits from PCa [4,5,6].

The presence of lymph nodes in the anterior periprostatic fat (APPF) and their significance were first reported in 2001 [9]. However, the same study concluded that the presence of metastasis in APPF lymph nodes is associated with poor prognosis; twenty-five years later, it remains a critical yet understudied aspect of surgical staging.

In our series, 18% of patients had lymph nodes present in APPF, almost the same as in a recent study from China [10]. This also aligns with historical data from two previous studies by Finley et al. [4], who reported a 14.7% incidence of lymph nodes in APPF, and Yuh et al. [5], who identified nodes in 16.7% of cases.

The number of lymph nodes in APPF varies, and ours is among the highest, whereas a 2012 study from the USA reported it as low as 0.8% [11]. We observed metastasis in 2.4% of patients in APPF LN, which corroborates an earlier study in which the malignancy rate was around 2.5% [5].

A striking observation was that some patients exhibited APPF metastasis despite negative pelvic nodes, a phenomenon previously documented [5,12]. This suggests that APPF nodes may represent an independent lymphatic drainage pathway distinct from the pelvic nodal basin, potentially bypassing conventional PLND templates. These findings underscore the limitations of relying solely on PLND for staging and advocate APPF evaluation to avoid underdetection of metastatic disease.

High-risk clinicopathological features were strongly associated with APPF metastasis. Among APPF-positive patients, 100% had MRI PIRADS ≥ 4, 100% had Gleason score ≥ 7, 100% had Briganti score > 20, and 58.3% had Briganti score > 50. These trends mirror prior studies linking APPF involvement to elevated PSA, advanced Gleason scores, and aggressive tumour biology [4,13]. Furthermore, the predominance of solitary APPF nodes (median = 1) in our cohort underscores the importance of meticulous pathological examination, as even a single node may upstage the disease.

Clinically, these results reinforce that APPF lymph nodes can harbour metastases even when pelvic nodes are negative. Omitting APPF analysis could underestimate disease extent in patients with otherwise unremarkable pelvic dissections. Routine excision of APPF in high-risk patients (Briganti score > 20) may therefore enhance staging accuracy, particularly if it alters the decision to pursue adjuvant therapies (e.g., radiation or systemic treatment). Our data further suggest that meticulous pathological evaluation of any APPF specimen is warranted, as missing even one positive node could affect prognosis and management. This aligns with the recommendations of Aning et al. [12], who advocate APPF removal as standard practice.

Despite the strength of a large cohort, this study is limited by its retrospective design and by the unavailability of all APPF histology samples, which may introduce selection bias. Future prospective studies could standardise APPF dissection protocols and correlate findings with long-term oncological outcomes, such as biochemical recurrence and survival. Multicentre research with uniform pathology assessment would further clarify the prognostic significance of APPF involvement.

## 5. Conclusions

This study emphasizes that lymph nodes within the APPF are not rare anatomical structures, but are clinically important and can contain metastatic disease, even if pelvic nodal involvement is not present. Routinely excising the APPF and analysing it histopathologically, especially in men with high-risk disease features, may help avoid underestimating nodal involvement and can inform better postoperative treatment decisions.

## Figures and Tables

**Table 1:** Analysis of patients with APPF Lymph node positivity to PSA, Gleason score, MRI, Stage, and Briganti score.

	Number of Patients	APPF Lymph Nodes Negative	APPF Lymph Nodes Positive
PSA			
5<	28	28	0 (0.0%)
5–10	38	33	5 (0.98%)
10–15	8	3	5 (0.98%)
15–20	4	2	2 (0.37%)
>20	2	2	0
Total	80	68	12 (2.3%)
Gleason Score (TRUS/Template)			
6	12	12	0 (0.0%)
7 (3 + 4)	34	31	3 (0.56%)
7 (4 + 3)	20	16	4 (0.78%)
8 or more	14	9	5 (0.98%)
Total	80	68	12 (2.3%)
MRI PIRAD			
3	10	10	0 (0.0%)
4	31	26	5 (0.98%)
5	39	32	7 (1.37%)
	80	68	12 (2.3%)
MRI Stage			
T2a	6	6	0 (0.0%)
T2b	12	12	0 (0.0%)
T2c	16	15	1 (0.19%)
T3a	23	18	5 (0.98%)
T3b	23	17	6 (1.17%)
	80	68	12 (2.3%)
Briganti Score			
Less Than 10	33	33	0 (0.0%)
10–20	19	19	0 (0.0%)
20–30	8	6	2 (0.37%)
30–40	7	5	2 (0.37%)
40–50	4	3	1 (0.18%)
50–60	4	2	2 (0.37%)
More Than 60	5	0	5 (0.98%)
	80	68	12 (2.3%)

## Data Availability

There was no new data created.
